# Prescribing Trends in Psychotropic Medications Among Outpatients of a Latin American Healthcare Setting: A Five-Year Retrospective Study

**DOI:** 10.7759/cureus.37832

**Published:** 2023-04-19

**Authors:** Jorge A Villalobos-Madriz, Bruno Serrano-Arias, Sebastián Arguedas-Chacón, Esteban Zavaleta-Monestel, Roberto Rodríguez-Miranda, José M Chaverri-Fernández, Alfredo Covarrubias-Gómez

**Affiliations:** 1 Department of Pharmacy, Hospital Clínica Bíblica, San José, CRI; 2 Department of Anesthesiology and Pain Management, Hospital Clínica Bíblica, San José, CRI; 3 Department of Pharmacology, Toxicology, and Pharmacodependency, Universidad de Costa Rica, San José, CRI; 4 Department of Pain and Palliative Medicine, National Institute of Medical Sciences and Nutrition Salvador Zubirán, México City, MEX

**Keywords:** hypnotics and sedatives, anticonvulsants, anti-anxiety agents, drug prescriptions, benzodiazepines, practice patterns, psychotropic drugs

## Abstract

Introduction

Mental health problems affect millions worldwide, and the prescription of psychotropic drugs is increasing globally. The World Health Organization (WHO) has emphasized the need for proper monitoring of psychotropic drug prescriptions. This study aims to characterize and find trends in the prescription of psychotropics in a Latin American General Hospital.

Methods

The study analyzed the dispensation of psychotropic prescriptions to outpatients at three pharmacies in the central headquarters of Hospital Clínica Bíblica in San José, Costa Rica, from 2017 to 2021. Psychotropic drugs were classified by the Anatomical Therapeutic Chemical (ATC) code, and the amount of each medication dispensed was standardized using the defined daily dose per 10,000 population per day metric. Patients' ages were categorized into four groups: under 18 years, 18 to 39 years, 40 to 64 years, and 65 years and above. The prescriptions were categorized according to medical specialty. Regression analyses were performed to determine the significance of trends observed in the data

Results

A total of 5793 psychotropic prescriptions were recorded. The average age of the patients was 58 years. The total consumption of psychotropics decreased by 33.94% from 2017 to 2021, with the most significant decline until 2020. However, there was an increase in consumption in 2021. Clonazepam was the most consumed medication, followed by bromazepam and alprazolam, which was the sole drug to exhibit an escalation in usage between 2017 and 2021. Regression analysis showed that only alprazolam and zopiclone had statistically significant trends. The highest number of prescriptions was dispensed to patients aged between 40 and 64 years, followed by those aged over 65 years. Anxiolytics were also the most commonly prescribed group of drugs. General medicine (20.22%), psychiatry (19.95%), and internal medicine (12.73%) were the primary specialties that prescribed psychotropic; 38.6% of prescriptions were associated with the 10th decile of patients, and 44.9% of prescriptions were issued by the 10th decile of physicians.

Conclusion

The consumption of psychotropic drugs decreased from 2017 to 2020 but increased in 2021, with alprazolam being the only drug that showed an increase in consumption throughout the entire period. General practitioners and psychiatrists were found to be the specialties that most commonly prescribe these medications. The study found significant trends only for the consumption of alprazolam and zopiclone and for prescription patterns among psychiatrists and internal medicine physicians.

## Introduction

Mental health problems have become a global public health concern. The high prevalence of mental health problems, estimated at 700 million people globally, continues to rise, presenting a significant challenge to health systems worldwide [1,2]. There is a need for increased attention and investment in mental health services to improve the well-being and functioning of individuals affected by mental health problems [1,3,4]. While pharmacological treatment is often used as the first-line treatment in countries where psychological interventions are difficult to access, there is still a substantial gap between the need for mental health treatment and its availability, especially in low-income and middle-income countries [4].

The prescription of psychotropic drugs has been increasing substantially worldwide, especially due to the high incidence of pathologies that require their use [4]. As a result, the World Health Organization (WHO) has made efforts to encourage health systems to initiate adequate surveillance of the prescription of psychotropics [5,6].

A psychotropic is a substance that acts on the central nervous system and can result in changes in behavior, alertness, mood, and consciousness [2,7,8]. Psychotropics include amphetamine derivatives, benzodiazepines, sedative-hypnotics, barbiturates, ketamine, and other central nervous system stimulants to control their prescription due to their influence on the nervous system [2,7,8].

Regarding the use of these drugs in the Latin American region, studies carried out in Argentina, Chile, and Colombia found an increase in their prescription and, worryingly, when characterizing the age groups that consume them, most of the medications were indicated for the elderly people who are commonly poly-medicated and are more likely to suffer adverse effects from pharmacotherapy [9-13].

Data from Costa Rica shows that the most prescribed psychotropics are benzodiazepines [5]. A report by the Costa Rican Drug Institute (Instituto Costarricense sobre Drogas (ICD)) in 2021 shows a stable trend in the consumption of these drugs, averaging 22,822 prescriptions dispensed monthly between 2018 and 2021 [14]. Interestingly, it also shows that its consumption was not increased statistically despite the psychiatric conditions triggered by the severe acute respiratory syndrome coronavirus 2 (SARS-COV-2) pandemic in this period [15-22].

Since the elderly are the population group that consumes most of the dispensed psychotropic drugs and considering that many of them are poly-medicated, leading to an increased risk of adverse effects and health complications, the characterization of the prescription of this drug group acquires relevance [7,19]. Additionally, given that the worldwide population is aging, an incorrect psychotropic prescription could directly affect the economy due to disabilities, adverse effects, and the increase in dependent or addicted patients [17,23].

Since 2016, the Ministry of Health of Costa Rica (MINSA) digitally registers the professionals and patients involved in the indication of controlled medications, providing traceability in the event of a case that needs to be analyzed [17,18].

This study aims to characterize and find trends in the prescription of psychotropics through the Digital Prescription platform in a Class A Latin American General Hospital in Costa Rica.

## Materials and methods

Study design

A retrospective and observational study was carried out from January 2017 to December 2021. Psychotropic prescriptions dispensed to outpatients from the three pharmacies of the central headquarters of Hospital Clínica Bíblica, located in San José, Costa Rica, were included. A total of 5793 psychotropic prescriptions were recorded [24]. As inclusion criteria, the prescriptions had to comply with having been prescribed and dispensed within the study period, and the medication in question needed to correspond to the group of psychotropics as defined by the Costa Rican General Health Law.

Data collection and analysis

The necessary information was obtained from electronic records (Digital Prescription) of the MINSA from a detailed report in a Microsoft Excel® file (Microsoft Corporation, Redmond, Washinton, United States) with the following data: prescription date, prescription number, purchase order, drugstore, income, balances, full name and professional code of the doctor, medical specialty, active ingredient, units dispensed, name and identification number of the patient; each document was divided by locality and respective year. Patients' ages were categorized into four groups: under 18 years, 18 to 39 years, 40 to 64 years, and 65 years and above. Prescriptions in which the doctor's specialty was not specified were excluded. Regarding physicians who had two or more specialties registered, their last specialty was the one considered. In addition, psychotropic medications that were not available on the market during the study period were excluded. The data were processed using Microsoft Excel.

Psychotropic drugs with the Anatomical Therapeutic Chemical (ATC) codes in various groups were included in the analysis, including antiepileptics (N03A), anxiolytics (N05B), and hypnotics and sedatives (N05C). The quantity of each drug dispensed was standardized using the defined daily dose per 10,000 population per day metric (DDDs/10,000/day), which is based on the defined daily dose (DDD) established by the WHO Collaborating Centre for Drug Statistics Methodology. The DDD corresponds to the estimated mean daily dose of a drug when used for its primary indication in adults and allows for easy comparisons of drug usage across different countries and formulations [25]. The population was defined as the inhabitants of the metropolitan area of Costa Rica, which consists of 2.6 million people.

Prescriptions were grouped according to medical specialty, comparing them with the total number of prescriptions processed. Additionally, the distribution of the number of prescriptions was obtained according to the age of the patients and vice versa, relating the age group to the prescribed medication. Finally, the data were processed to calculate the decile of patients according to the number of prescriptions, and in the same way to relate the prescribers with the number of prescriptions to characterize the prescription.

Regression analyses were performed to determine the significance of trends observed in the data using Microsoft Excel. The built-in regression analysis tool was utilized to fit quadratic, and cubic polynomial regression models to the data. Additionally, the p-values associated with the coefficients of the regression models were calculated to assess the significance of the trends, with a p-value less than 0.05 being considered statistically significant.

Ethical considerations

This study was approved in session No. 260 by the Ethics Committee of the Universidad de Costa Rica (approval number: CEC-284-2022). This study did not require written consent.

## Results

**Figure 1:**
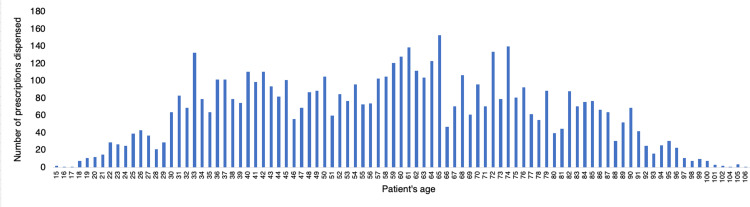
Total psychotropic prescriptions dispensed by patient's age

In the studied period, 5793 prescriptions of psychotropic medications were dispensed to 2602 patients who met the criteria for the analysis. These prescriptions were made by 609 distinct physicians. Figure 1 shows the total number of prescriptions dispensed according to the age of the patients. The distribution of prescriptions by age exhibited a normal pattern, with patients ranging from 15 to 106 years old and an average age of 58.

**Figure 2:**
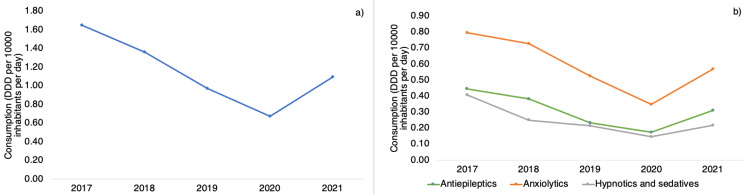
(a) Overall psychotropic consumption among outpatients per year, (b) Trends in psychotropic consumption per ATC classification ATC: Anatomical Therapeutic Chemical

The total consumption of psychotropics decreased from 1.65 DDD/10,000/day in 2017 to 1.09 DDD/10,000/day in 2021, indicating a lowering of 33.94%. Figure 2 illustrates an overall decreasing trend in usage that was more prominent until 2020. However, in 2021, there was an upward trend in consumption, which was the only year to do so. The same trends can be seen when classifying drugs by ATC code. Anxiolytics were the group of drugs most used in the period studied. Even so, its consumption went from 0.79 DDD/10,000/day to 0.57 DDD/10,000/day. Antiepileptics, which only includes the use of clonazepam within this category, decreased its use from 0.45 DDD/10,000/day to 0.31 DDD/10,000/day. Finally, hypnotics and sedatives dropped from 0.41 DDD/10,000/day to 0.22 DDD/10,000/day. The statistical analysis did not reveal significant results for the overall trend (p=0.164), as well as for anxiolytics (p=0.315), antiepileptics (p=0.175), and hypnotics and sedatives (p=0.053).

**Figure 3:**
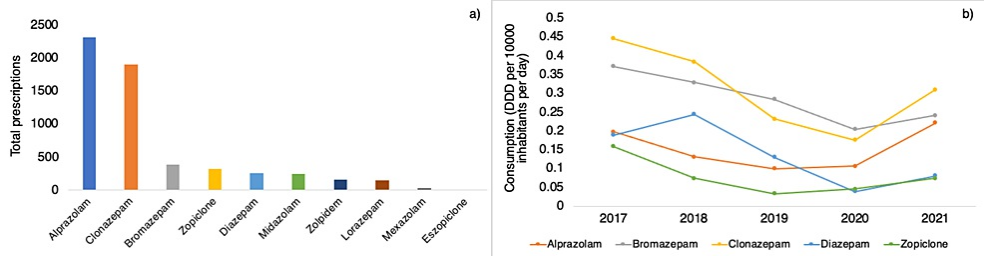
(a) Total prescriptions according to psychotropic medication; (b) Trends in psychotropic consumption among outpatients per medication

The consumption of medications and the number of prescriptions by active ingredient are presented in Figure 3. The most consumed medication in the studied population was clonazepam, followed by bromazepam and alprazolam. Among the medications, alprazolam had the highest count of prescriptions and was the sole drug to exhibit an escalation in usage between 2017 and 2021, rising from 0.20 DDD/10,000/day to 0.22 DDD/10,000/day. During the statistical analysis, only alprazolam (p=0.025) and zopiclone (p=0.010) showed significant results among all the individual drugs tested. The other commonly used drugs, such as bromazepam (p=0.391), clonazepam (p=0.175), and diazepam (p=0.991), did not exhibit statistically significant trends.

**Figure 4:**
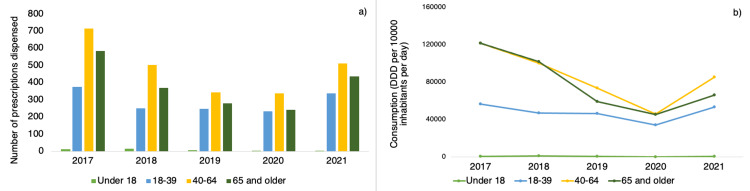
(a) Total prescriptions of psychotropic medication dispensed by age group; (b) Trends in psychotropic consumption among outpatients by age group

The outcomes of patient age classification are presented in Figure 4. The highest number of prescriptions in the study period were dispensed to patients aged between 40 and 64 years, with those aged over 65 years coming in second. The consumption patterns in adults followed the trends mentioned earlier. There were no statistically significant trends observed for patients under 18 years (p=0.999), those between 18 to 39 years (p=0.239), those between 40 to 64 years (p=0.201), and those aged 65 years and above (p=0.160).

**Figure 5:**
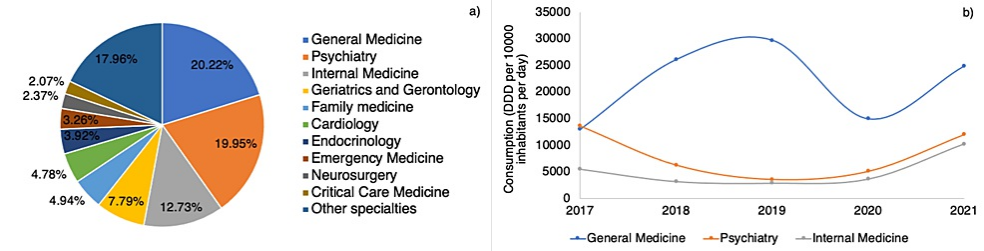
(a) Distribution of psychotropic prescriptions according to medical specialty; (b) Trends in psychotropic consumption among outpatients by medical specialty

A total of 51 different medical specialties prescribed psychotropics in the studied period, as shown in Figure 5. The main prescribing specialties were general medicine (20.22%), psychiatry (19.95%), and internal medicine (12.73%). The prescription patterns for the three physician types were as follows: General practitioners showed an increase in prescription rate from the start of the study period until 2020. However, the trend for this group of physicians was not statistically significant (p=0.532). Conversely, psychiatrists (p<0.001) and internal medicine specialists (p=0.036) showed significant trends in prescription rates, with an increase in consumption from 2020 onwards.

**Figure 6:**
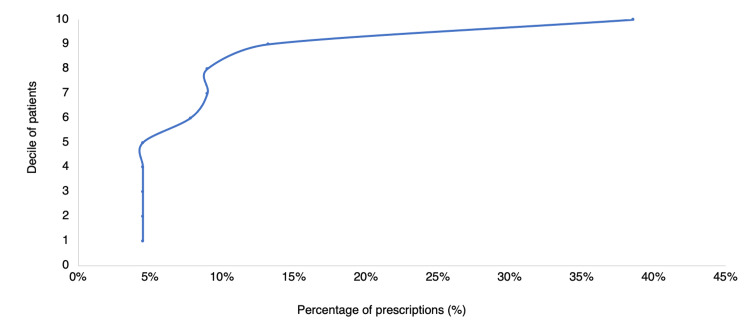
Percentage of psychotropic prescriptions according to the patient decile

**Figure 7:**
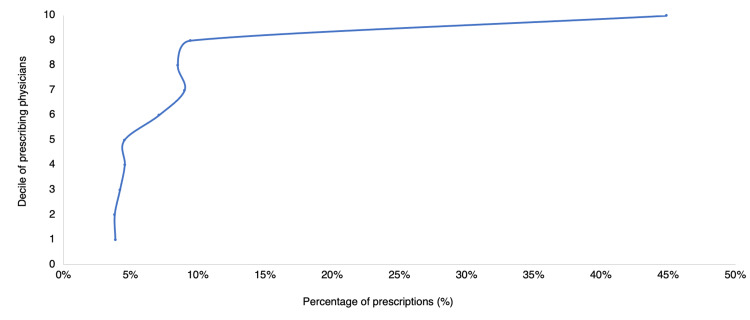
Distribution of prescriptions according to the decile of the prescribing physician

Upon analyzing the deciles for patients and prescriptions in Figure 6, it was observed that 38.6% of prescriptions were associated with the 10th decile. Meanwhile, Figure 7 demonstrated that 44.9% of prescriptions were issued by the 10th decile of physicians.

## Discussion

According to the findings of this study, the use of psychotropic drugs exhibited a fluctuating pattern over time. While there was a decline in consumption between 2017 and 2020, there was a subsequent increase in usage after that point. However, the overall consumption of these medications in 2021 did not reach the levels observed in 2017, except for alprazolam. These results deviate from the general patterns observed in other studies conducted globally [1,4], such as one in China which reported an upward trend in the use of psychotropic drugs from 2018 to 2021 [1]. It is worth noting that high-income countries demonstrated a greater increase in psychotropic drug use compared to upper-middle and lower-middle-income countries [4].

An important topic that marked the time of this study, which could be a factor in the increase in the use of psychotropics in 2021, was the coronavirus disease 2019 (COVID-19) pandemic. Various studies have mentioned the behavior of the consumption of this type of medication since the start of the pandemic and the isolation measures that were taken in a large number of countries. The pandemic presented a high epidemiological burden of depression, anxiety disorders, stress, and many more mental health problems [26].

One study found an increase during the pandemic in the prescriptions of psychiatric drugs, which increased by 6.1%, highlighting the higher consumption of antidepressants, anxiolytics, and sedative-hypnotic medications in women, older people, and rural areas [27]. Another study in Portugal found significantly increasing trends in the prescription of anxiolytics, sedatives, and hypnotics reported among women aged 65 years or above [28].

The findings of this study can be compared with other research conducted in the country. For example, a study on the use of psychotropic drugs in Costa Rica during the pandemic found that while there were no significant differences in drug consumption, they did observe similar patterns to those described in this paper. Specifically, they noted a decrease in drug use in April 2020 followed by a peak in March 2021 [14]. According to another study, clonazepam is the most prescribed psychotropic medication in the country. Despite being ranked third in terms of the number of prescriptions, it is the most widely consumed psychotropic medication among outpatients at the hospital [29].

When evaluating the age groups, the average age of patients for each active ingredient is around 58 years. This highlights a predictable point, given that sufficient international bibliography shows that the population that mostly consumes psychotropics is the elderly [5,6]. Even though the elderly were not the group with the highest consumption of these drugs, they are a significant group of patients who rely on these medications, and this population represents a growing percentage of the Costa Rican population over time.

Another important point when analyzing these results is the impact on public health. Depending on their dosage, benzodiazepines can cause drowsiness and dizziness, and in general, can depress the central nervous system, that in turn can cause secondary complications such as increased fall risks, hip fractures, traffic accidents, and others [19]. It is known that these events can directly affect the quality and life expectancy in these cases, which is why it is important that doctors, pharmacists, and healthcare providers, in general, educate patients and caregivers to obtain the maximum benefit from these medications without falling into excessive use or prescription [19].

When analyzing the results shown in Figure 5, almost 50% of the total prescriptions were made by general practitioners and psychiatrists. This is related to another study carried out in Brazil, which found that these are the two specialties that most commonly prescribe these drugs [30]. As expected, psychiatry comes as one of the main prescribing specialties; however, it is essential to highlight that it is general medicine that has a majority of prescriptions, which shows how crucial it is to generate educational strategies or campaigns for the rational use of psychotropics and, on the other hand, how the pharmacist professionals must be able to assess the need for treatment and suggest de-escalation to reduce the irrational use of these drugs. Correlating the average age of the patients studied with the main prescribing specialties, it is natural to find geriatricians in fourth place on this list.

Except for specific cases like alprazolam and zopiclone, most of the patterns did not yield statistically significant results but did exhibit comparable tendencies, especially an increase in consumption from 2021. Further research into the prescription and use of these drugs is necessary to determine whether factors such as the COVID-19 pandemic are significant determinants.

By reviewing the number of prescriptions in each patient, it is shown in Figure 6 that in terms of public health, the fact that the number of prescriptions is distributed in this way by deciles of patients indicates that the majority of people who used psychotropics in the period studied did not have a prolonged dispatch in the time since it is observed that the 10th decile is the one that houses 38.6% of the prescriptions, giving a minority of patients with a large number of prescriptions. In the same way, it is worrying that the patient with the highest number of prescriptions obtained 52 in four years; however, this represents a particular case and does not speak for most of the population.

Similarly, as in another study related to the use of narcotic drugs, analyzing the distribution of the number of prescriptions by decile of physicians (Figure 7), it is noted that around 44.9% of the prescriptions are focused on the 10th decile [20-24]. This can be related to the fact that the pharmacies under this study belong to a general hospital with an outpatient service, so it is expected that there will be a minority of doctors who concentrate a large amount of the total prescription of psychotropics.

The most apparent flaw in the psychotropic medication policy and system utilized in Costa Rica presently is the absence of an obligation to specify the patient's diagnosis while prescribing. Although it is possible to include the diagnosis in the prescription notes, it is not a common practice. Requiring the diagnosis to be mentioned would simplify the task of pharmacists in verifying the appropriateness of the medication selection, dosage, and treatment duration. There are occasional inconsistencies that can be detected when utilizing this system, such as incorrect patient ages or weights that do not match their actual values.

One of the strengths of this study is its ability to provide evidence regarding the present utilization of psychotropic medication in the Latin American region, where there is a lack of reporting on this type of medication. Although the Digital Prescription System of Costa Rica has functioned effectively since its implementation, the inclusion of the diagnosis in prescriptions would enhance the dispensing process, making it more comprehensive and secure.

The present study presented several limitations since the data were obtained only from a private healthcare center, which does not necessarily reflect the national reality. The extracted information did not include certain information such as the doctor-patient relationship and adherence to treatment or diagnosis, and so the prescriptions were not qualitatively characterized. Although the norm is to present the normalized results in DDD/1000/day, in this study they are presented in DDD/10,000/day to make the results more presentable because the values found were very small.

## Conclusions

The use of psychotropic drugs showed a fluctuating pattern over time, with a decline in consumption between 2017 and 2020 and a subsequent increase in usage after that point. However, the overall consumption of these medications in 2021 did not reach the levels observed in 2017, except for alprazolam. General practitioners and psychiatrists were found to be the specialties that most commonly prescribe these medications. It is important to educate patients and caregivers about the rational use of psychotropics and to assess the need for treatment to reduce the irrational use of these medications. In terms of consumption patterns, statistical significance was only observed for alprazolam and zopiclone. As for the prescription trends according to medical specialty, significant trends were only observed among psychiatrists and internal medicine physicians.
